# Editorial: Translating biomechanics of the human airways for classification, diagnosis and treatment of pulmonary diseases

**DOI:** 10.3389/fphys.2025.1743792

**Published:** 2025-11-24

**Authors:** Aranyak Chakravarty, Saikat Basu, Mahesh V. Panchagnula, Neelesh A. Patankar

**Affiliations:** 1 School of Nuclear Studies and Application, Jadavpur University, Kolkata, India; 2 Department of Applied Mechanics and Bio-medical Engineering, Indian Institute of Technology Madras, Chennai, India; 3 Department of Mechanical Engineering, South Dakota State University, Brookings, SD, United States; 4 Department of Mechanical Engineering, Northwestern University, Evanston, IL, United States

**Keywords:** airway biomechanics, airway mucus, aerosol deposition, asthma, COPD, virtual disease landscape, aerosolisation, lung infections

Pulmonary diseases remain a global health challenge, with chronic obstructive pulmonary disease (COPD), asthma, pneumonia, tuberculosis, and lung cancer accounting for millions of deaths annually. The COVID-19 pandemic further underscored the critical need for mechanistic understanding of respiratory function and pathophysiology. While clinical insights have advanced substantially, the biomechanics of the human airways offer an underexplored yet promising route to early diagnosis and effective therapy. Airway biomechanics operates at multiple scales—ranging from the microscale mechanics of mucus and cilia to the macroscale deformation of the bronchial tree during respiration. Translating these multiscale phenomena into diagnostic or therapeutic insights requires cross-disciplinary collaboration between physiologists, engineers, and clinicians. This Research Topic brings together recent advances that bridge the gap between fundamental biomechanics of the human airways and clinical translation of biomechanical knowledge through computations and experimental studies. The articles in this Research Topic discuss novel treatment strategies for COPD and asthma, pulmonary drug delivery and ventilation strategies, inter-personal morphological variability, mucociliary clearance, infection progression and aerosol formation within the respiratory tract.

Treatment strategies for COPD and asthmatic patients is a critical issue faced by pulmonologists across the world. The article by Li et al. sheds light on how deeper insights into pathogenesis and phenotyping of COPD may be obtained using a combination of single-photon emission computed tomography (SPECT) and quantitative computed tomography (qCT)-derived biomarkers. These insights can be utilised to develop new avenues for evaluating COPD progression and devising appropriate therapeutic responses. A different interventional approach was adopted by Abu Shaphe et al. They investigated how treadmill exercise responses, guided by the 6-min walk test, vary among individuals with differing COPD severity. Their findings suggest that personalising treadmill exercise protocols according to COPD severity levels and walk test results can reveal important functional limitations and help tailor rehabilitation strategies for improved outcomes in COPD management. The study by Zhong et al. show that Fe_2_O_3_ nanoparticles – when used in optimal concentration – can potentially disrupt the microstructure of airway mucosal fluid significantly lowering the viscoelastic nature of the mucosal fluid and facilitating easier mucus clearance from airways. Tests using mucus from asthmatic patients confirm these findings suggesting that Fe_2_O_3_ nanoparticles could act as expectorants, potentially outperforming conventional mucolytics by virtue of their biocompatibility and availability.

Pulmonary drug delivery presents a plausible route for achieving efficient systemic drug delivery (West, 2012). However, despite the clinical relevance, its efficiency remains suboptimal owing to significant deposition of inhaled aerosolised drug dose in the upper respiratory tract (Bessler and Sznitman, 2024). Compensation through larger doses is often prescribed although undesirable side effects or even systemic exposure is possible (De Boer et al., 2017). Various novel strategies are, thus, being investigated for overcoming this drawback (Dua et al., 2020; Chakravarty et al., 2022; Kole et al., 2023). Bessler et al. reports on a relatively less investigated strategy – leveraging the inherent electrostatic charge present on inhaled aerosols. Their study – using an *in vitro* airway-on-chip platform mimicking small bronchial geometries – reveal that electrostatic forces substantially alter deposition patterns in constricted airways: for submicron particles, there is enhanced proximal airway deposition due to electrostatic-diffusive effects, while larger particles show extended deposition footprints beyond what gravity alone would allow. These results suggest electrostatic attraction could be strategically used to improve the targeting of inhaled therapeutics in obstructive lung diseases like asthma and COPD.

The pulmonary drug delivery systems also warrant personalisation for achieving desired therapeutic efficacy due to inter-patient variability in lung morphology. Direct quantification of such variability is, however, not possible beyond the seventh lung generation owing to technological limitations, despite recent advances in imaging and diagnostic techniques. The article by Karthiga Devi et al. discusses a novel non-invasive method to estimate morphology of the distal lung by measuring radio-aerosol deposition patterns in healthy individuals utilizing gamma scintigraphy. The results demonstrate that aerosol deposition, particularly in the distal airways, serves as a sensitive marker of morphological variability, suggesting this approach could be developed into a walk-in lab test to personalize diagnosis and optimize pulmonary drug delivery.

A different aspect of personalised respiratory care is highlighted in Vijay Anand et al. They presented a mechanistic compartmental model designed to investigate how airway secretion accumulation and its removal affect respiratory dynamics in ICU patients on mechanical ventilation. The study identifies characteristic changes in ventilator waveforms due to secretion buildup—such as reduced inspiratory flow and longer exhalation—and introduces a model-informed secretion index for continuous bedside monitoring. These findings can be utilised to improve personalized respiratory care and secretion management for mechanically ventilated patients.

Another key contribution to this Research Topic (Chakravarty et al.) discusses the development of a versatile physics-based, 1D reduced-order computational model encompassing varied and complex bio-physical phenomena involving airflow, gas exchange, particle/aerosol transport and deposition, mucus transport and pathogen infection progression within the human airways. The model has been utilised for identifying routes of improving drug delivery to the acinar region of the lung (Chakravarty et al., 2022). A different application of this model (Chakravarty et al., 2023) led to the hypothesis of re-aerosolisation of nasopharyngeal mucosa (RNM) as a plausible route of COVID-19 infection spread to the acinar region of the lung (Morawska et al., 2022), while reinforcing the utility of vaccination in preventing fatal infections.

The hypothesis of RNM is being investigated through computations and experiments by various groups (Pairetti et al., 2021; Anzai et al., 2022; Kant et al. 2023; Saha et al., 2024; Li et al., 2025). One such group (Ilegbusi et al.) studied the formation and transport of mucus droplets during cough across CT-derived upper airway geometries. Increases in mucus thickness and viscosity—characteristic of respiratory diseases—is observed to substantially affect the number and size of exhaled droplets, with thicker mucus yielding more and larger droplets, while more viscous mucus results in fewer but larger droplets. Similar findings were reported by Saha et al. (2024). A more generic case of the same mechanism involving inhaled lower airway transmission of pathogen-laden microdroplets fragmented from the upper airway mucosa has been recently explored in a different study (Basu, 2025), through full-scale computational simulation of the intra-airway inhalation physics within CT-based anatomical domains. The simulated patterns of advective transport were validated against reduced-order analytical estimates (tracking the impact of dominant vortex cores in the laryngotracheal space on downwind bronchial transport) and published experimental results (Miguel, 2017). The mechanism has been hypothesized (Basu, 2025; Chakravarty et al., 2023) as a key factor for brisk onset of secondary deep lung infections, following the emergence of symptoms in the upper respiratory tract. These findings show that the distribution of exhaled cough droplets can act as a sensitive, non-invasive biomarker for classifying pulmonary diseases, underlining the diagnostic potential of droplet dynamics and mucosal properties in respiratory health assessment.

To summarise, the article Research Topic on the Research Topic highlights the potential of airway biomechanics to be used for defining clinically-relevant metrics called physiomarkers and develop diagnostic tools. For example, the reduced-order model (Chakravarty et al.) introduces several dimensionless parameters for defining lung physiology which can be used to const*ruc*t a virtual disease landscape (VDL) -- essentially a mapping of different disease groups and normal function in a hyperspace of dimensionless parameters controlling lung function. These parameters defining the VDL can help obtain mechanistic insights into pathologies and hence used as physiomarkers for disease classification and diagnosis. The VDL – coupled with machine learning techniques – could be developed into a powerful diagnostic tool for disease trajectory prediction, optimize therapeutic interventions, and ultimately improve patient outcomes.
